# Supplementary material for: Chromatin accessibility landscape and regulatory network of high-altitude hypoxia adaptation
Source: Nat Commun. 2020 Oct 1;11:4928. doi: 10.1038/s41467-020-18638-8 (PMC7529806; doi:10.1038/s41467-020-18638-8)
Supplement: Supplementary file 1 — Supplementary Information [file 41467_2020_18638_MOESM1_ESM.pdf]

Supplementary Information for

Chromatin accessibility landscape and regulatory network of high-altitude hypoxia adaptation

Jingxue Xin[1,2,3,4,5]*, Hui Zhang[1,3]*, Yaoxi He[1,3,5]*, Zhana Duren[2,6,7]*, Caijuan Bai[8]*, Lang Chen[2,5], Xin Luo[1,3,5], Dong-Sheng Yan[9], Chaoyu Zhang[2,5], Xiang Zhu[6], Qiuyue Yuan[2,5], Zhanying Feng[2,5], Chaoying Cui[8], Xuebin Qi[1,3], Ouzhuluobu[8], Wing Hung Wong[4,6,7]†, Yong Wang[2,3,5,10]†, Bing Su[1,3]†

†To whom correspondence should be addressed.
Email: sub@mail.kiz.ac.cn (B.S.); ywang@amss.ac.cn (Y.W.); whwong@stanford.edu (W.H.W.)

* These authors contributed equally to this work.

This PDF file includes:

Supplementary Figures 1-16

Captions for Supplementary Figures 1-16

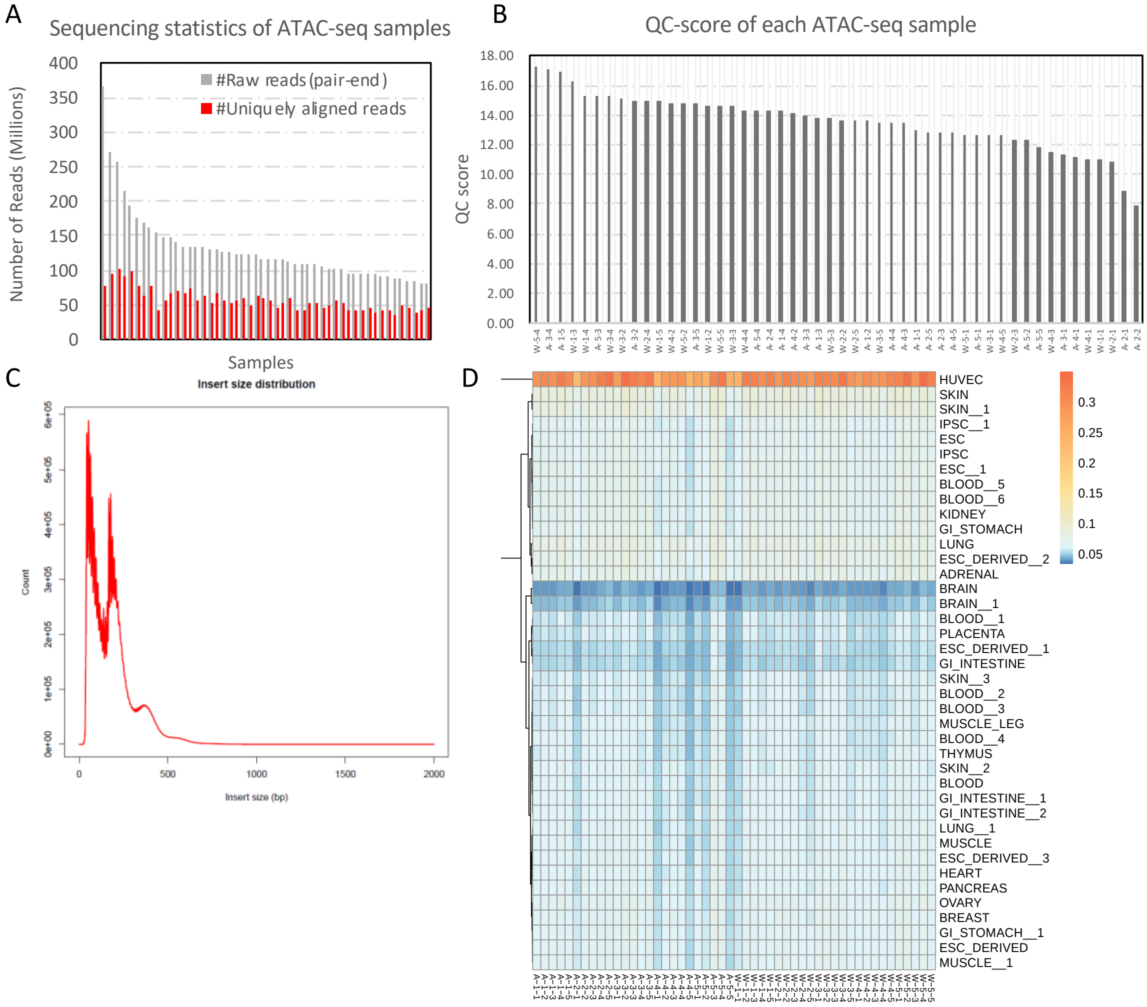

Supplementary Figure 1. Quality check for the 50 ATAC-seq data by their sequence depth, fragment distribution, QC score, and overlap with the public dataset. (A) The number of raw reads and percentile of uniquely aligned reads. The number of raw reads ranges from 80 to 260 Million for each ATAC-seq sample, and the percentiles of uniquely aligned reads across all samples is 45% on average. (B) QC scores of all ATAC-seq samples. QC score is defined as the ratio of total reads count at TSS centered up- and down-stream 2-kb window to the randomly selected background [-3k,-2k] among all genes. All the samples reach the standard score 8, i.e., the fold change is large than 2, which shows the ATAC-seq signals are enriched in open chromatin regions such as promoters. (C) Insert size distribution of one example W-1-1. The other 49 samples show a similar pattern. This fragment length distribution reveals a sharp peak at less than 100bp regions for nucleosome-free fragments, the second large peak is within 200bp for the mono-nucleosome fragment, and more other peaks, which indicate good data quality. (D) Correlations between 50 ATAC-seq samples in our hypoxia induction experiment and 40 DNase-seq samples from ROADMAP. Correlation is calculated as the Jaccard similarity between two open region lists, i.e., the total length of overlap regions divided by the length of merged regions. Fro all 50 samples, the public HUVEC

DNase sample (~30% similarity) ranks the first compared with other tissues. Source data are provided as a Source Data file.

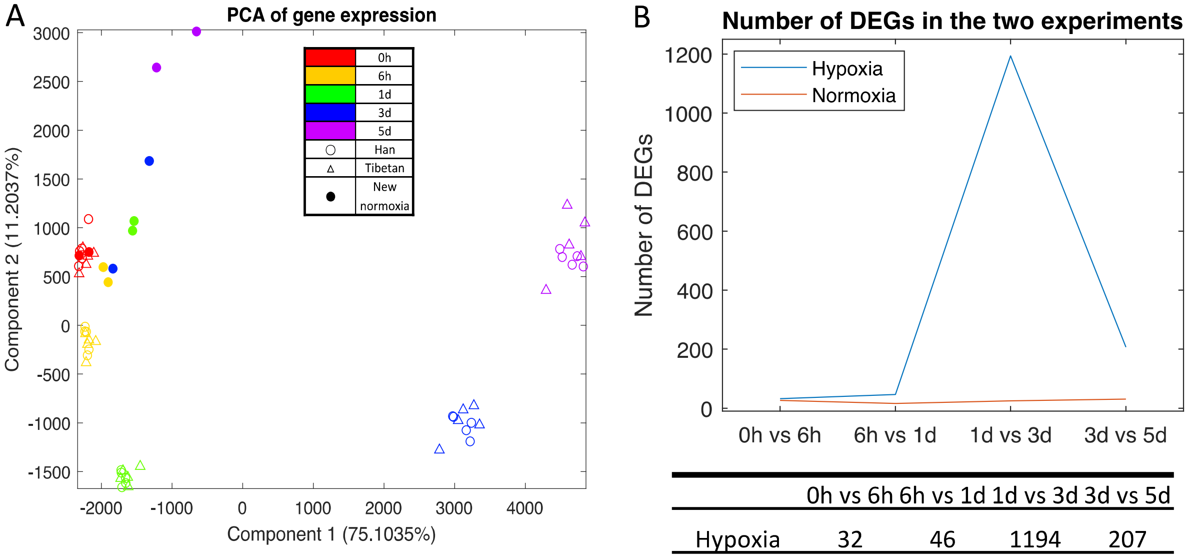

	0h vs 6h	6h vs 1d	1d vs 3d	3d vs 5d
Hypoxia	32	46	1194	207
Normoxia	26	16	25	31

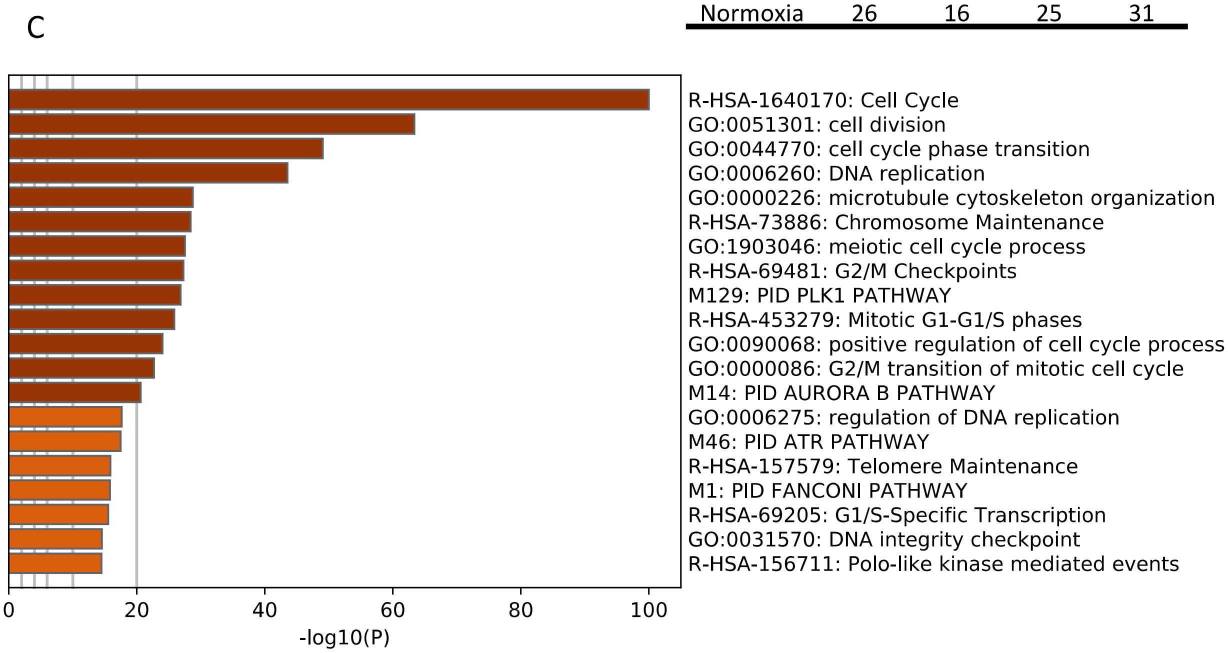

Supplementary Figure 2. Expression profiles of HUVECs cultured in normoxia for five days reveal responses to being in culture for different periods of time are insignificant and negligible compared to

the responses to hypoxia. (A) PCA of gene expression data of HUVECs cultured in normoxia and hypoxic conditions for 5 time points. After batch effect adjustment, the normoxia samples (solid circles) are close to the hypoxia 0h samples (red samples). The largest variance revealed by PC1 is the response to hypoxia. The variance between normoxia samples is relatively much smaller than the hypoxia ones (hollow shapes), especially in 3d and 5d (PC2 11% vs PC1 75%). (B) Comparison of the number of DEGs between adjacent time points in normoxia and hypoxia experiments. Red line indicates normoxia, while blue line represents hypoxia. The numbers of DEGs in hypoxia response, especially 1d vs 3d and 3d vs 5d, are significantly larger than in normoxia by more than 30 folds (1,000 vs 30). (C) Functional enrichment of 560 DEGs (Fold change >2) between 0h and 5d for the response to cell culture along time. The functional terms are significantly enriched in cell cycle and DNA replication ($p\text{-value}<10^{-40}$, hypergeometric test with Benjamini-Hochberg correction), which are not related to hypoxia response.

A

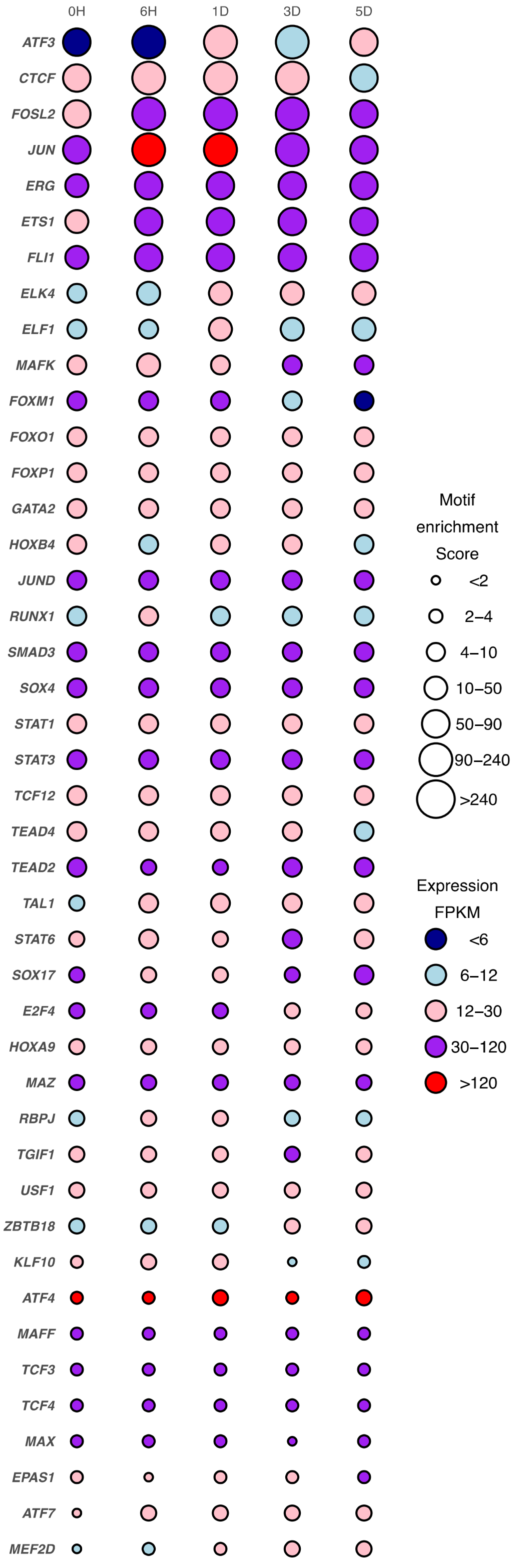

B

Pathway name	LogP	Hits
angiogenesis	-5.4924793	EPAS1,ETS1,JUN,TAL1,SOX17,MEF2D, STAT6,MAFK
blood vessel morphogenesis	-5.1109563	EPAS1,ETS1,JUN,TAL1,SOX17
blood vessel development	-4.7752706	EPAS1,ETS1,JUN,TAL1,SOX17
vasculature development	-4.6967949	EPAS1,ETS1,JUN,TAL1,SOX17
cardiovascular system development	-4.6655574	EPAS1,ETS1,JUN,TAL1,SOX17
erythrocyte differentiation	-4.5803807	EPAS1,ETS1,TAL1
erythrocyte homeostasis	-4.4773466	EPAS1,ETS1,TAL1
response to hydrogen peroxide	-4.251318	ETS1,JUN,STAT6
response to oxidative stress	-4.0730066	EPAS1,ETS1,JUN,STAT6
response to reactive oxygen species	-3.519417	ETS1,JUN,STAT6
cellular response to oxidative stress	-3.4251181	EPAS1,ETS1,STAT6

Supplementary Figure 3. Key TFs response to hypoxia by motif enrichment in chromatin accessibility peaks and dynamic expression levels. (A) Key TFs response to hypoxia. We listed top TFs based on their expression level (FPKM) and motif enrichment score (the production of -log10 p-value and fold change of each motif in each ATAC-seq peak list, p-values are calculated by Homer) along 5 time points. The motif scores are divided into 7 levels and shown by circle size, while the expression FPKM is grouped into 5 levels with various colors. (B) Functional terms related to angiogenesis that are enriched by TFs in **Fig. 1D**. P-values are computed by hypergeometric test with Benjamini-Hochberg correction. Source data are provided as a Source Data file.

A

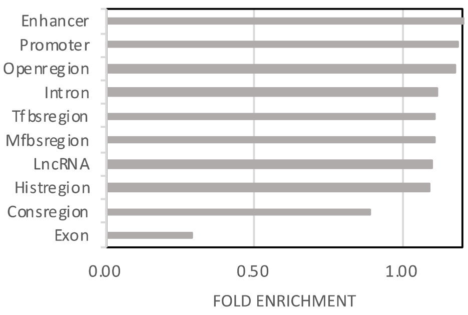

Functional region	SNP count	Ratio	Enriched p-value
Exon	823	0.29	1
Consregion	7,774	0.89	1
Histregion	111,182	1.09	0
LncRNA	37,207	1.10	5.97E-108
Mfbsregion	98,536	1.11	0
Tfbsregion	107,356	1.11	0
Intron	50,736	1.12	1.07E-230
Openregion	75,308	1.18	0
Promoter	2,360	1.19	1.12E-16
Enhancer	19,487	1.22	1.89E-182

B

C

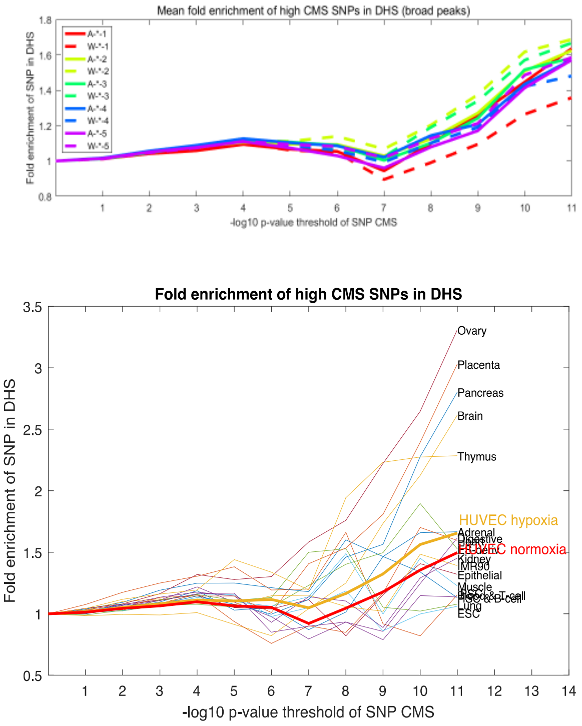

Supplementary Figure 4. SNPs under selection in Tibetan are enriched in open regions of HUVEC and functional tissues of multiple tissues. (A) 111,182 SNPs under selection (FDR<0.05) are enriched in annotated regulatory regions such as enhancers and promoters in ENCODE. For each functional region set, we calculated the fold enrichment score of selection SNPs in these regions. The below table listed the fold ratio and enrichment p-value. P-value is calculated using a binomial distribution with the distribution function (**Methods**). (B) Selected SNPs are enriched in peaks for all 50 ATAC-seq samples. The x-axis is the threshold of CMS score (defined as $-\log_{10}$(p-value), where p-values were calculated by Fisher's method) that measures the selection status, and the y-axis denotes the fold enrichment computed by the ratio, defined as the number of SNPs above a certain threshold to all SNPs (threshold is 0) normalized by

region length (**Methods**). Each color refers to a time point (1-0h, 2-6h, 3-1d, 4-3d, and 5-5d), and solid lines are Tibetan (Adaptive (A)) samples, while dash lines are Han Chinese (Wildtype (W)). The sample name in legend box means population-*-time, for example, A-*-1 means the average fold enrichment score of all 5 adaptive samples in 0h. (C) SNPs under selection are highly enriched in HUVEC and a variety of cell types/tissues from ROADMAP. The FC score is calculated across 40 DNase-seq tissues from ROADMAP (**Methods**). More stringent thresholds give higher fold enrichment scores. Source data are provided as a Source Data file.

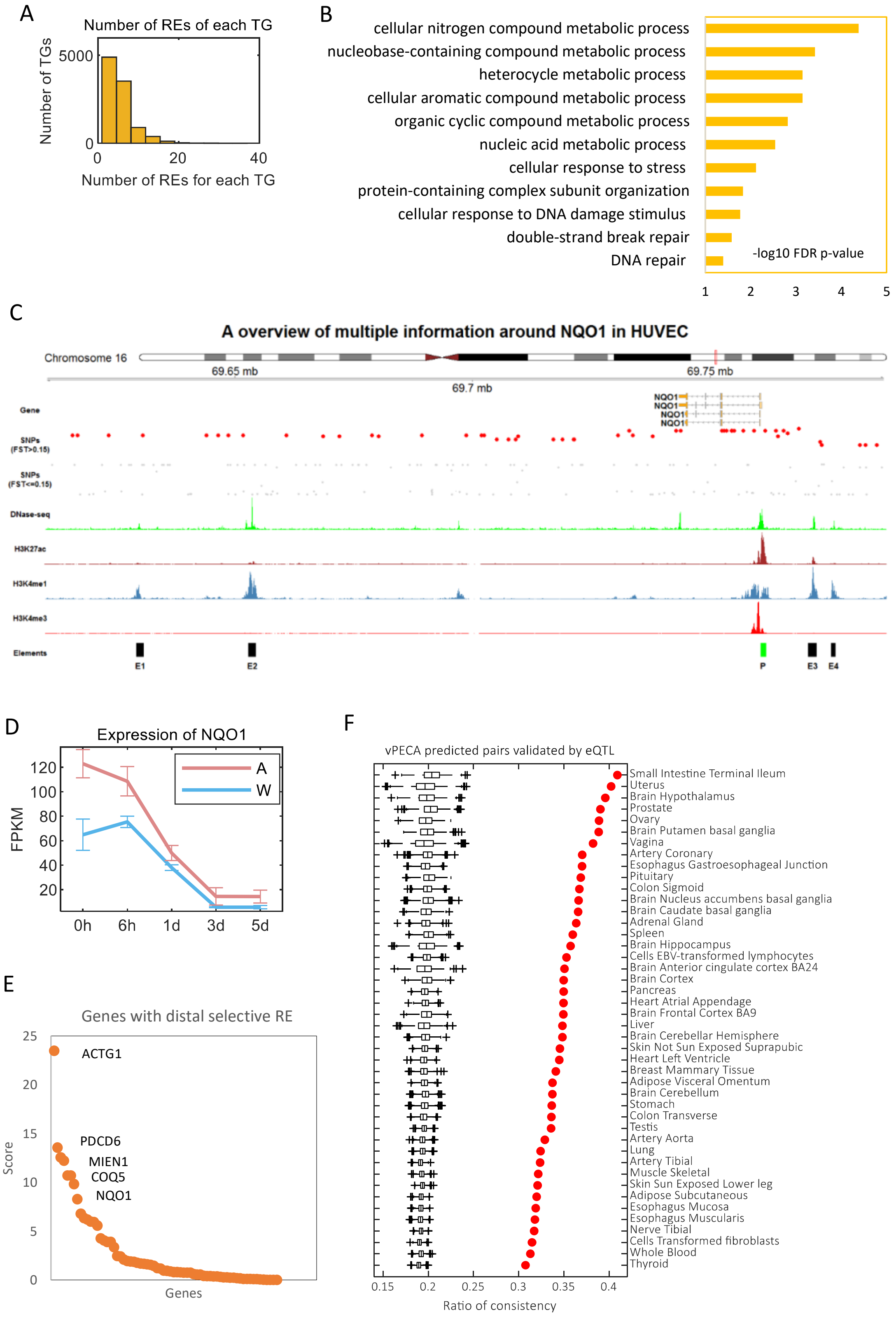
A
Number of REs of each TG
Number of TGs
5000
0
0
20
40
Number of REs for each TG
B
cellular nitrogen compound metabolic process
nucleobase-containing compound metabolic process
heterocycle metabolic process
cellular aromatic compound metabolic process
organic cyclic compound metabolic process
nucleic acid metabolic process
cellular response to stress
protein-containing complex subunit organization
cellular response to DNA damage stimulus
double-strand break repair
DNA repair
-log10 FDR p-value
1
2
3
4
5
C
A overview of multiple information around NQO1 in HUVEC
Chromosome 16
69.65 mb
69.7 mb
69.75 mb
Gene
NQO1
SNPs (FST>0.15)
SNPs (FST<=0.15)
DNase-seq
H3K27ac
H3K4me1
H3K4me3
Elements
E1
E2
P
E3
E4
D
Expression of NQO1
FPKM
120
100
80
60
40
20
A
W
0h
6h
1d
3d
5d
E
Genes with distal selective RE
Score
25
20
15
10
5
0
ACTG1
PDCD6
MIEN1
COQ5
NQO1
Genes
F
vPECA predicted pairs validated by eQTL
Small Intestine Terminal Ileum
Uterus
Brain Hypothalamus
Prostate
Ovary
Brain Putamen basal ganglia
Vagina
Artery Coronary
Esophagus Gastroesophageal Junction
Pituitary
Colon Sigmoid
Brain Nucleus accumbens basal ganglia
Brain Caudate basal ganglia
Adrenal Gland
Spleen
Brain Hippocampus
Cells EBV-transformed lymphocytes
Brain Anterior cingulate cortex BA24
Brain Cortex
Pancreas
Heart Atrial Appendage
Brain Frontal Cortex BA9
Liver
Brain Cerebellar Hemisphere
Skin Not Sun Exposed Suprapubic
Heart Left Ventricle
Breast Mammary Tissue
Adipose Visceral Omentum
Brain Cerebellum
Stomach
Colon Transverse
Testis
Artery Aorta
Lung
Artery Tibial
Muscle Skeletal
Skin Sun Exposed Lower leg
Adipose Subcutaneous
Esophagus Mucosa
Esophagus Muscularis
Nerve Tibial
Cells Transformed fibroblasts
Whole Blood
Thyroid
0.15
0.2
0.25
0.3
0.35
0.4
Ratio of consistency

Supplementary Figure 5. Validation of vPECA predicted RE-TG regulations. (A) Distribution of the number of REs for each TG. Most of the genes have a small number of REs. About 5,000 genes have less than four REs associated with them. Only a few genes have dozens of REs. (B) Functional enrichment of selected REs with differential accessibility in local time points. Functions such as stress response and metabolic process rank at the top. P-values were calculated by hypergeometric test with Benjamini-Hochberg correction. (C) The regulatory map for *NQO1* shows many weakly selected SNPs are located in REs. (D) NQO1's dramatic expression changes along time point and the down-regulation pattern. Data are presented as mean values +/- standard error (n=5). (E) Detection of new candidate genes under selection based on distal RE. Among vPECA predicted selected RE and TG pairs, we selected those REs with Pr(S=1)>0.99 and at least contains one selection SNP (Fst>0.1). Then identified distal RE, i.e., the distance between RE and TSS is longer than 10-kb. Finally, those genes that are not identified by current studies are listed. The genes are ranked by their functional score, defined as the following formulation. FS=$\log_{10}$ (p of DEG)×log10 (p of DO) ×(Gene expression)×(Openness of RE)×(RE-TG correlation). *NQO1* is one interesting gene related to oxidoreductase. (F) vPECA predicted RE-TG pairs are validated by eQTL. eQTL data in 44 tissues from the GTEx database are used to validate vPECA predicted pairs. Sensitivity, i.e., the number of predicted pairs overlap with eQTL divided by the total number of eQTL supported pair, is calculated as a measurement to compare two randomly selected background. The blue null distribution is a random selection of the same number of REs nearby expressed genes [-300k,+300k] for 1,000 times. The orange distribution is selected from the blue one by following the distance distribution of vPECA predicted RE-TG pairs. The sensitivity of vPECA predicted pairs (red dots) are significantly higher than randomly selected ones across all tissues (black boxplot). Boxplots are represented by minima, 25% quantile, median, 75% quantile, and maxima (n=1000). Source data are provided as a Source Data file.

A

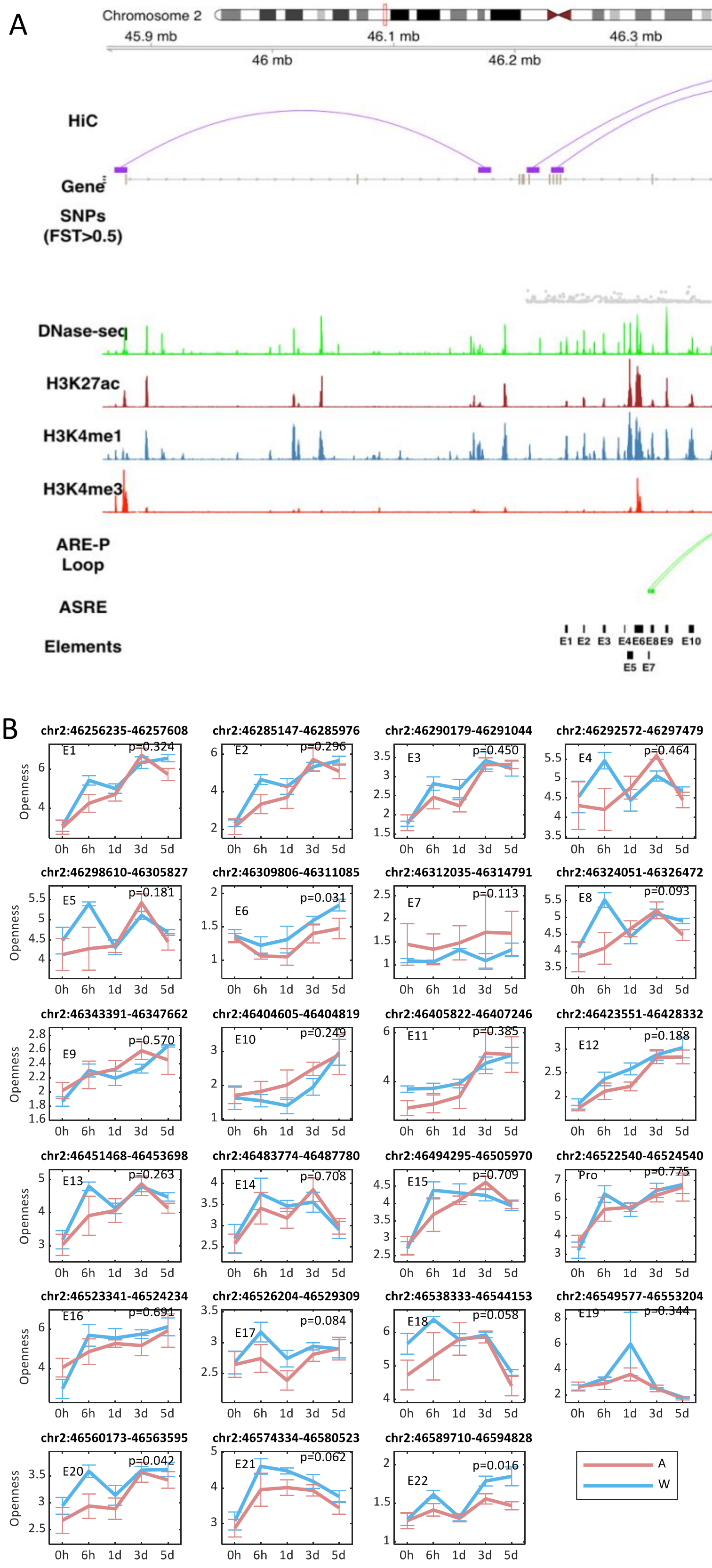

C

$$TG = \beta_{-1} + \sum_{i=0}^{m} \beta_i O_i + \varepsilon;\ \varepsilon \sim N(0, \sigma^2)$$

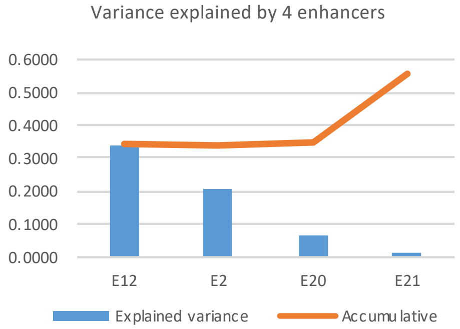

	Estimate	Pr(>|t|)
R square	0.5560	
Intercept	5.7309	0.5431
E2	-0.1335	0.9221
E12	20.3198	0
E20	2.7520	0.4299
E21	-9.9000	0

D

$$TG = \beta_{-1} + \sum_{i=0}^{m} \beta_i Z_i \left(\sum_{k \in MB_i} B_{i,k} \cdot TF_k \right) + \varepsilon$$

$$\varepsilon \sim N(0, \sigma^2)$$

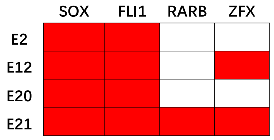

	Estimate	Pr(>|t|)
R2	0.681521	
(Intercept)	5.168938	0.339864
E2	-0.00423	0.496073
E12	0.08239	3.42E-05
E20	0.034821	0.039251
E21	-0.04419	7.60E-06

E

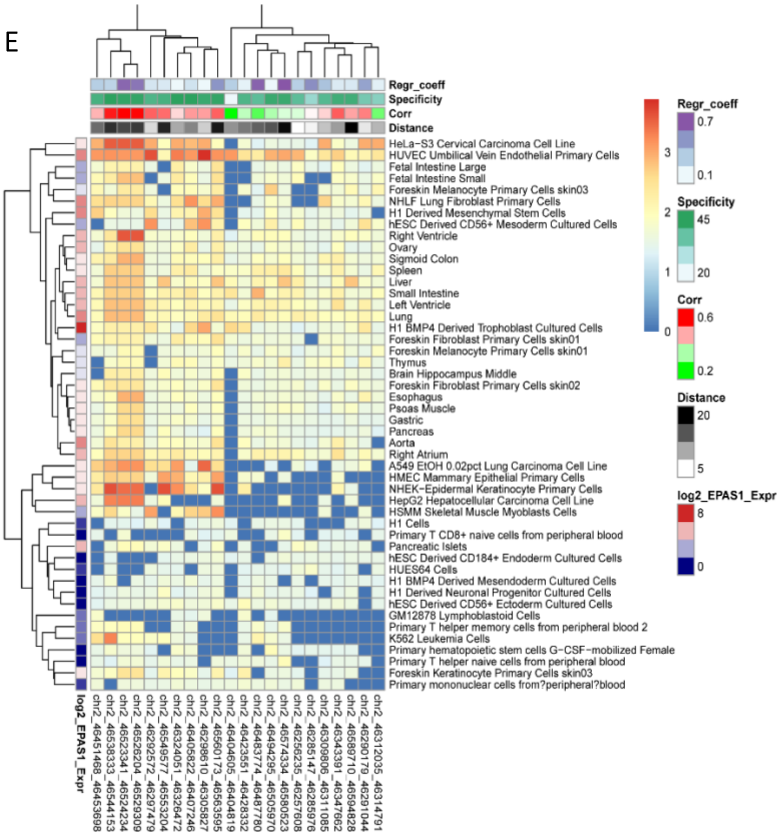

Supplementary Figure 6. The chromatin accessibility dynamics of *EPAS1*'s REs and regulatory role revealed by linear regression. (A) The regulation landscape of *PRKCE* and *EPAS1*. (B) The chromatin accessibility dynamics of all 23 *EPAS1*'s REs. Red lines indicate Tibetans and blue lines are Hans. P-value was calculated by two-sided t-test (n=25). Data are presented as mean values +/- standard error (n=5). (C) Explained variance of four REs selected by linear regression of enhancer openness. Blue bars refer to the explained variance of *EPAS1* expression by each RE independently, and the red line represents accumulative explained variance by the combination of REs. E12 gains 34% variance and E21 complements another 20% variance. (D) Adding 4 TFs binding to the REs increases the R-square of linear regression from 55% to 68%. (E) H3K27ac signals of 23 REs across 48 tissues in ROADMAP. The color bar demonstrates the maximal fold change of H3K27ac peaks overlap with each RE. The column bar "log2_EPAS1_Expr" shows EPAS1 expression in each tissue. The row "Regr_coeff" is the regression coefficients of RE's H3K27ac signals to gene expression, while the "Corr" row gives the cross tissue correlation between the H3K27ac signal of each RE and gene expression. RE specificity cross tissues is defined as the number of tissues that RE is active (overlap with H3K27ac peaks), and is shown in the "Specificity" indicator bar. Generally, higher *EPAS1* expression tissues tend to have more active REs. vPECA predicted active REs E12 and E21 are clustered together. Source data are provided as a Source Data file.

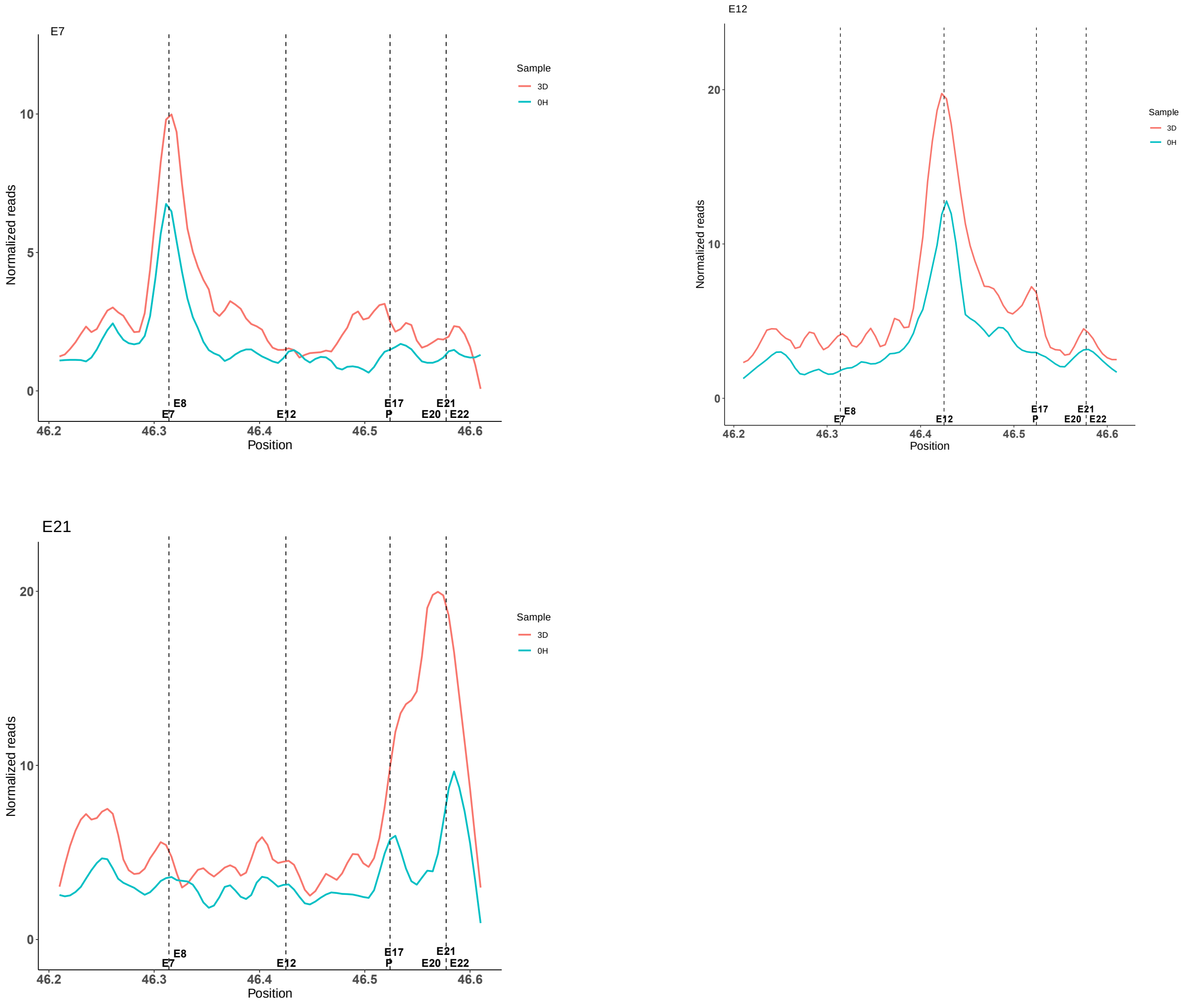

Supplementary Figure 7. The HiC data for *EPAS1*'s active REs E7, E12, and E21. They all show RE-promoter's interaction as the strongest signal.

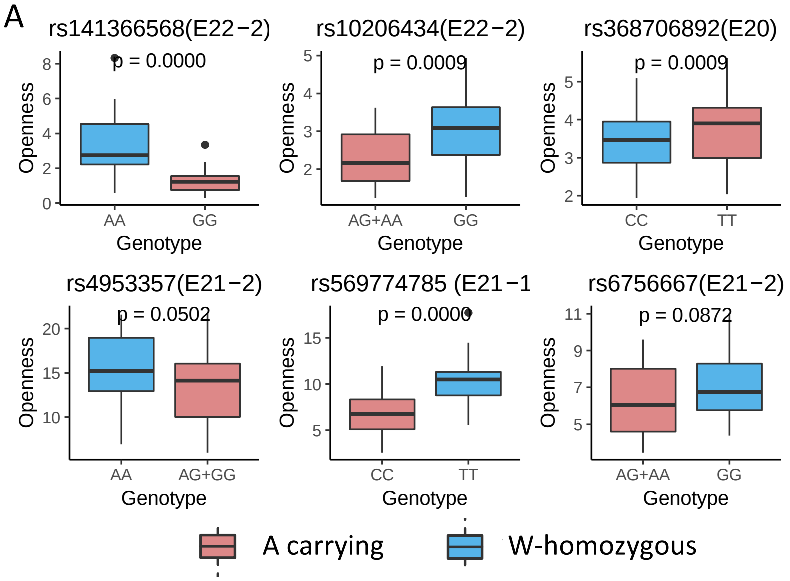

NAME	SHORT ENHANCER	LENGTH	SNP	HAN	TIB
E12-1	chr2:46424139-46424664	526			
E12-2	chr2:46427352-46427884	533			
E2	chr2:46285207-46285760	554			
E20	chr2:46561271-46561457	186	rs370299814	G	A
E20	chr2:46561577-46561727	150	rs368706892	C	T
E21-1	chr2:46577457-46577909	452	rs569774785	T	C
E21-2	chr2:46578939-46579448	509	rs4953357	A	G
E21-2	chr2:46578939-46579448	509	rs6756667	G	A
E22-1	chr2:46593447-46593666	219	rs10206434	G	A
E22-2	chr2:46593993-46594186	193	rs141366568	A	G
E22-1	chr2:46591848-46592282	434	rs3768729	C	T

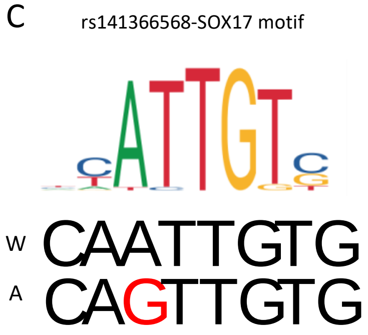

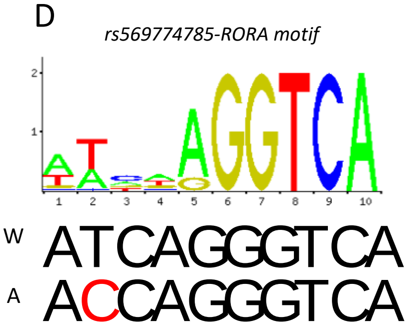

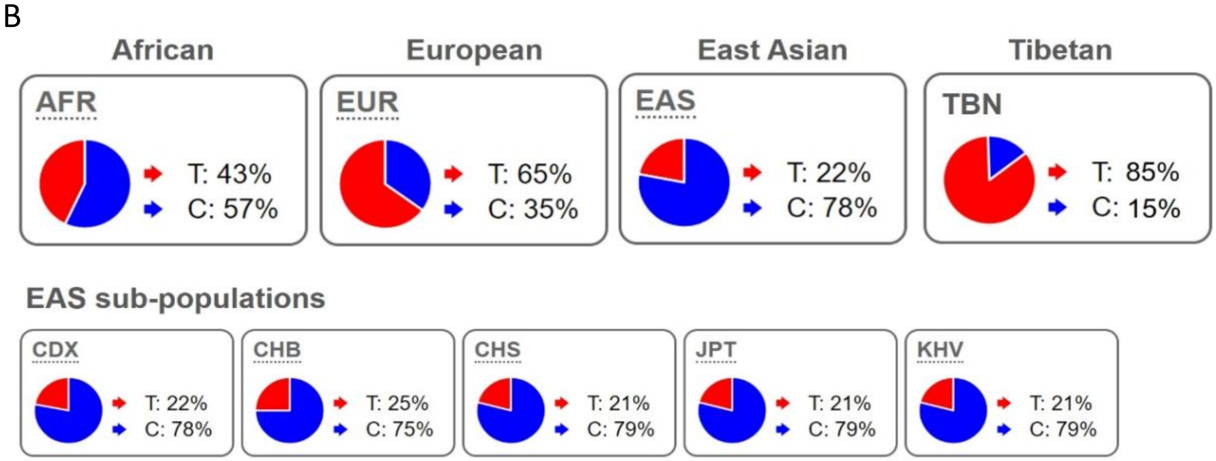

E **HUVEC**

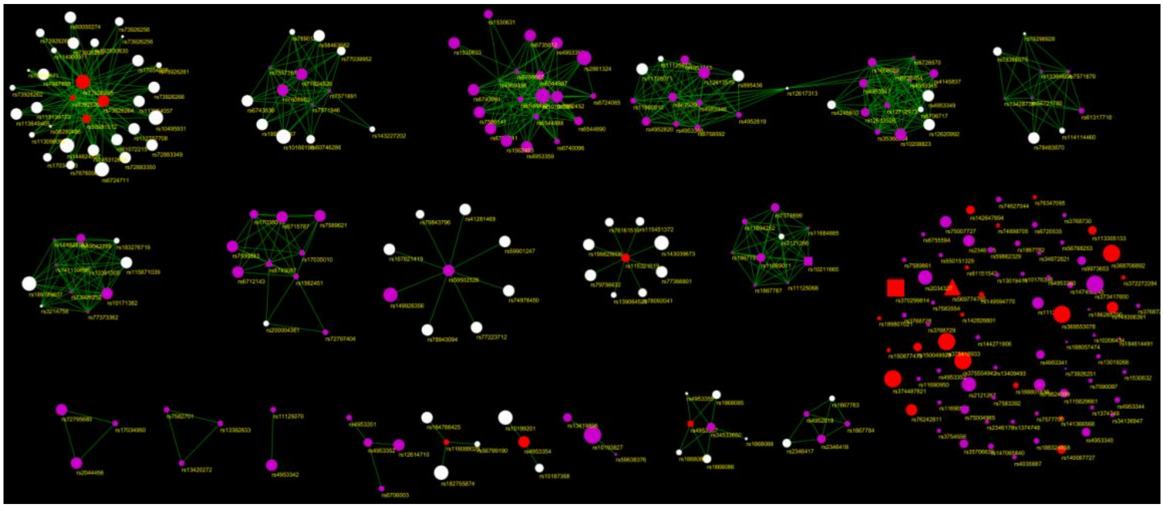

ESC

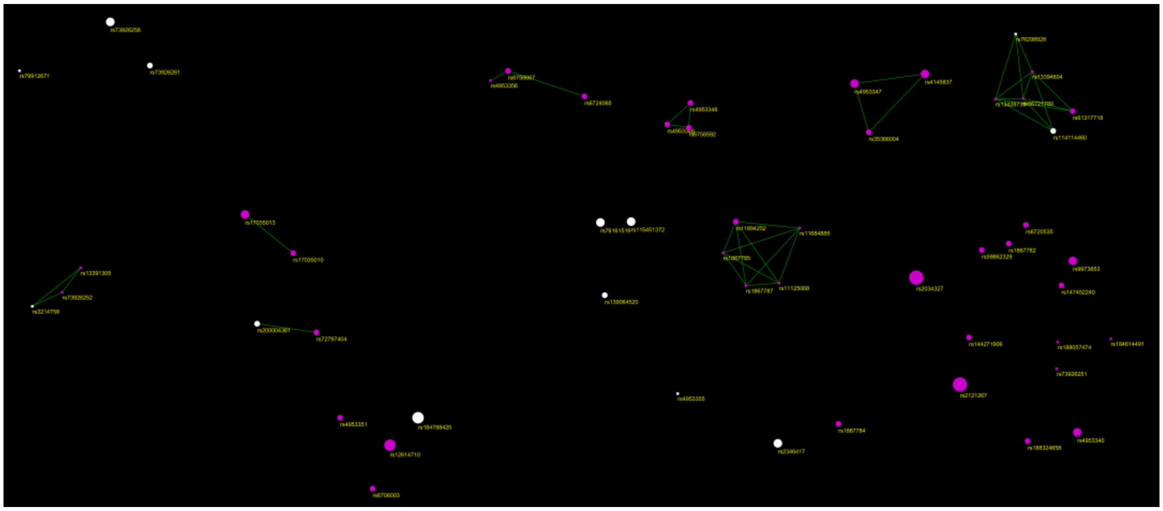

F

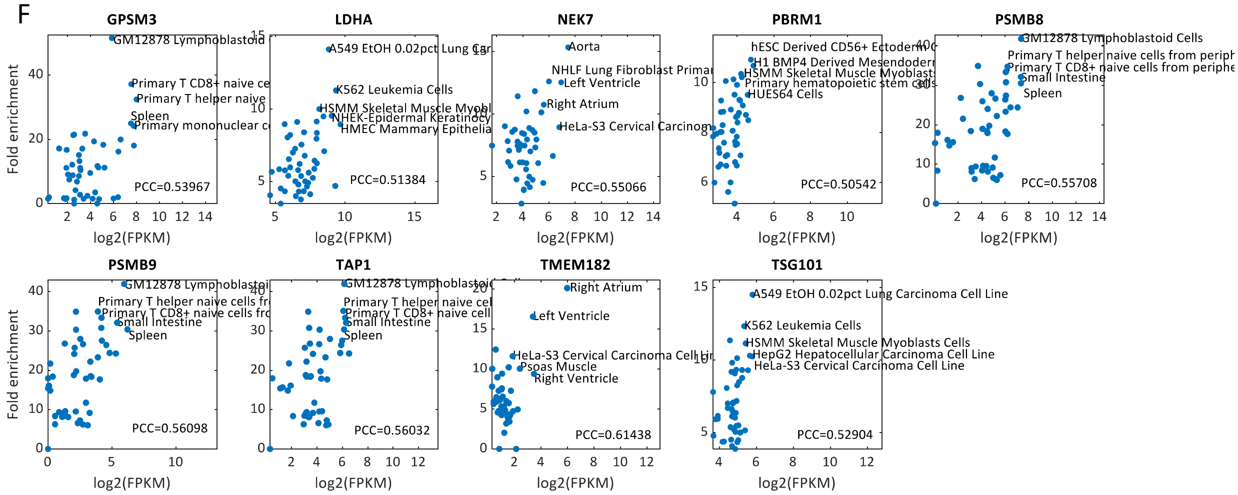
GPSM3
LDHA
NEK7
PBRM1
PSMB8
PSMB9
TAP1
TMEM182
TSG101
Fold enrichment
log2(FPKM)
PCC=0.53967
PCC=0.51384
PCC=0.55066
PCC=0.50542
PCC=0.55708
PCC=0.56098
PCC=0.56032
PCC=0.61438
PCC=0.52904
GM12878 Lymphoblastoid Cells
Primary T helper naive cells from peripheral blood
Primary T CD8+ naive cells from peripheral blood
Small Intestine
Spleen
A549 EtOH 0.02pct Lung Carcinoma Cell Line
K562 Leukemia Cells
HSMM Skeletal Muscle Myoblasts Cells
HepG2 Hepatocellular Carcinoma Cell Line
HeLa-S3 Cervical Carcinoma Cell Line
Right Atrium
Left Ventricle
Psoas Muscle
Right Ventricle
Aorta
NHLF Lung Fibroblast Primary Cells
NHEK-Epidermal Keratinocyte Primary Cells
HMEC Mammary Epithelial Primary Cells
hESC Derived CD56+ Ectoderm Cultured Cells
H1 BMP4 Derived Mesendoderm Cultured Cells
Primary hematopoietic stem cells
HUES64 Cells
Primary mononuclear cells from peripheral blood

Supplementary Figure 8. EPAS1's SNPs under positive selection and their LD linkages network. (A) SNPs under selection in EPAS1's active REs show differentially opened signals between two populations. The openness value is calculated within a 100bp bin around each SNP. Red bar indicates the samples of all time points with adaptive carrying genotype, while blue ones represent all wildtype homozygous samples. All SNPs selected as experiment candidates are differentially opened between two genotypes measured by t-test. Three SNPs rs141366568, rs10206434, and rs569774785 are significantly different with p-value $< 10^{-3}$ (n=25 samples, one-sided t-test). Boxplots are represented by minima, 25% quantile, median, 75% quantile, and maxima. (B) Allele frequencies of rs3768729 in global populations in 1000 Genomes (Phase 3). CDX: Chinese Dai in Xishuangbanna, CHB: Han Chinese in Beijing, CHS: Southern Han Chinese, JPT: Japanese in Tokyo, and KHV: Kinh in Ho Chi Minh City. (C) SNP rs141366568 may change the motif binding strength of *SOX17*. The adaptive allele G will weaken the binding score compared with wildtype. (D) Adaptive allele C of rs569774785 may weaken the binding strength of *RORA*. (E) SNP LD network in HUVEC (up) and ESC (down). 180 SNPs are selected in the body of *EPAS1*, among them 23 red SNPs are recognized as high Fst SNPs (Fst>0.5) and other 167 SNPs are in purple. The SNPs located in the open regions (DHS) are shown. If two SNPs have LD r2>0.8, there is an edge between them (white dots represent other SNPs in LD of 180 SNPs but not in them). There are more SNPs in open chromatin regions of HUVEC than in ESC, and the high Fst SNPs tend to be in a large LD region. (F) The number of selected SNPs in REs is positively correlated with gene expression levels across tissues. 9 genes with PCC>0.5 are listed. The fold change is defined as the number of high Fst SNPs per kb in context active region. Source data are provided as a Source Data file.

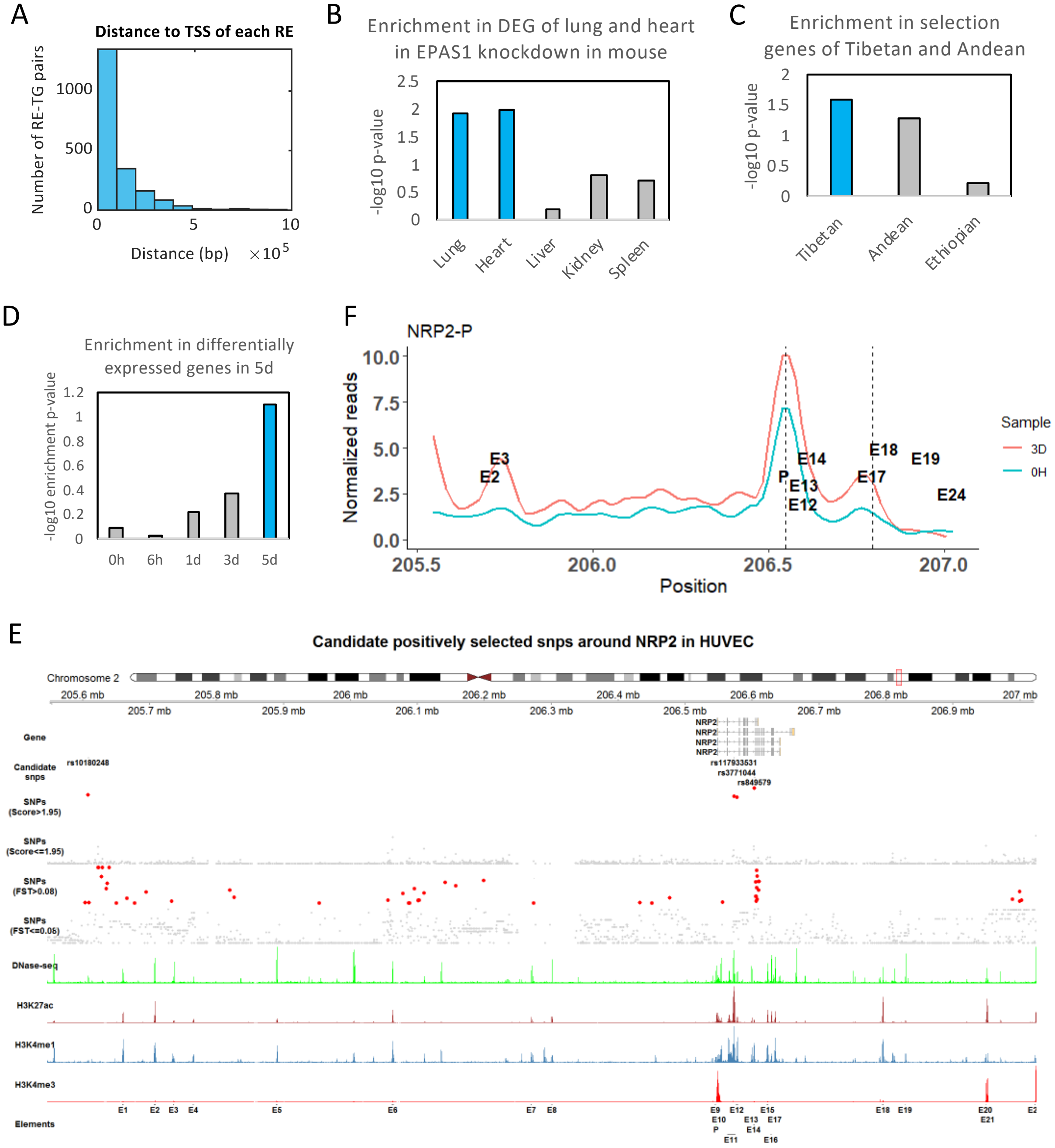

Supplementary Figure 9. **Validation of *EPAS1*'s downstream 621 TGs**. (A) The distribution of the distance between TG and RE in *EPAS1*'s downstream subnetwork. Almost all REs are within the 300-kb region from TSS. (B) TGs of *EPAS1* are enriched in DEGs (two-sided t-test FDR p<0.05 between knock-out and control) of lung and heart in heterozygous *EPAS1* knock-out experiments in mouse [1]. P-values (hypergeometric test) are 0.018 (37 overlap) and 0.010 (30 overlap). (C) 621 TGs tend to be positively selected in Tibet and the Andean population (hypergeometric test p-value 0.025 (25 overlap) and 0.087 (17 overlap)). Genes under selection in the two populations are searched from [2]. (D) 621 TGs tend to be enriched in differentially expressed genes in d5 after hypoxia pressure. DEGs at each time point are calculated by t-test between 5 Hans and 5 Tibetans. Genes with p-value <0.05 are identified. (E) Regulatory map for *NRP2*.

(F) *NRP2* promoter and enhancer regulations are validated by HiC's chromatin interaction data between REs and promoter. Source data are provided as a Source Data file. Source data are provided as a Source Data file.

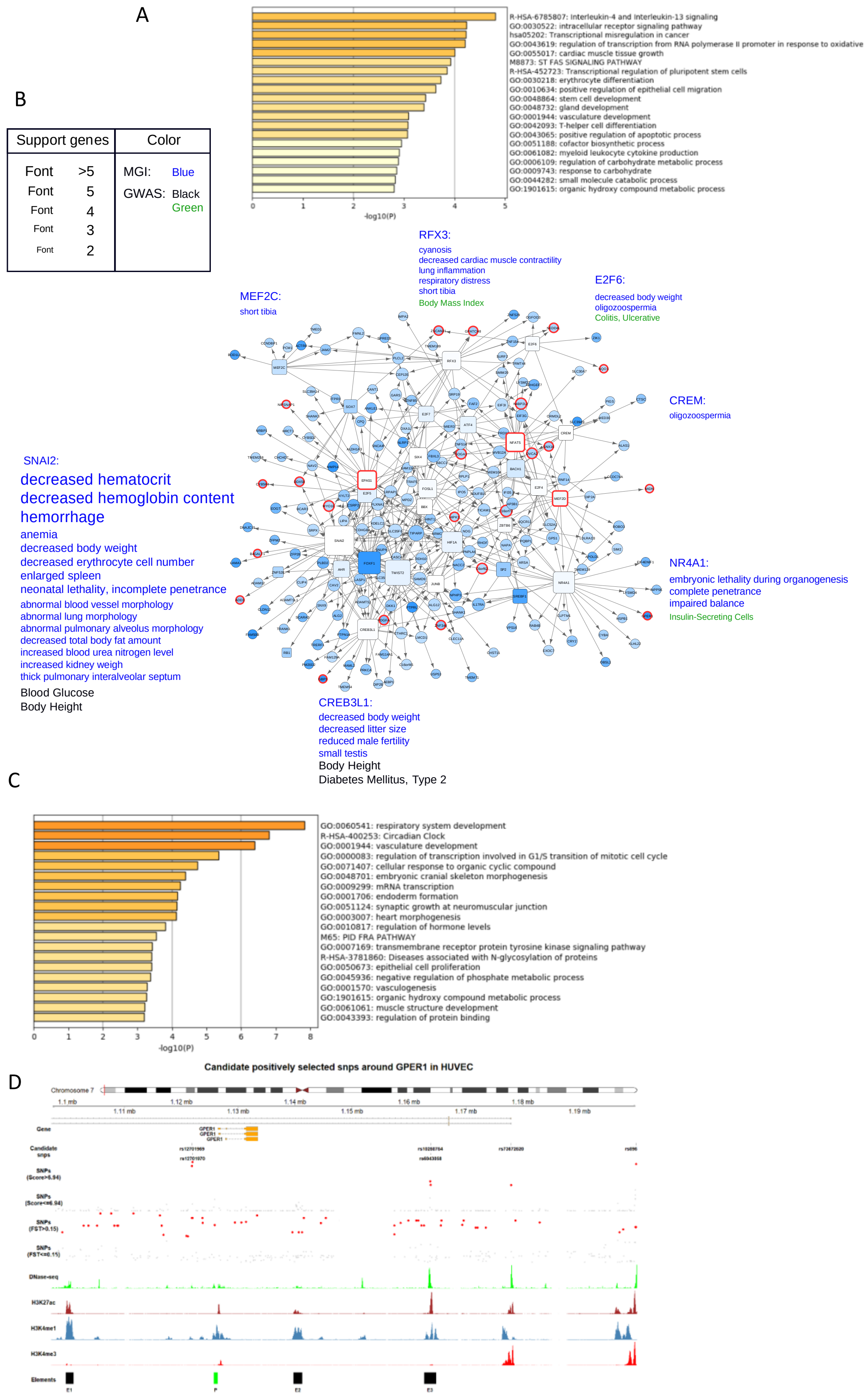
A
R-HSA-6785807: Interleukin-4 and Interleukin-13 signaling
GO:0030522: intracellular receptor signaling pathway
hsa05202: Transcriptional misregulation in cancer
GO:0043619: regulation of transcription from RNA polymerase II promoter in response to oxidative
GO:0055017: cardiac muscle tissue growth
M8873: ST FAS SIGNALING PATHWAY
R-HSA-452723: Transcriptional regulation of pluripotent stem cells
GO:0030218: erythrocyte differentiation
GO:0010634: positive regulation of epithelial cell migration
GO:0048864: stem cell development
GO:0048732: gland development
GO:0001944: vasculature development
GO:0042093: T-helper cell differentiation
GO:0043065: positive regulation of apoptotic process
GO:0051188: cofactor biosynthetic process
GO:0061082: myeloid leukocyte cytokine production
GO:0006109: regulation of carbohydrate metabolic process
GO:0009743: response to carbohydrate
GO:0044282: small molecule catabolic process
GO:1901615: organic hydroxy compound metabolic process
0 1 2 3 4 5
-log10(P)
B
Support genes
Color
Font >5
Font 5
Font 4
Font 3
Font 2
MGI: Blue
GWAS: Black
Green
RFX3:
cyanosis
decreased cardiac muscle contractility
lung inflammation
respiratory distress
short tibia
Body Mass Index
E2F6:
decreased body weight
oligozoospermia
Colitis, Ulcerative
MEF2C:
short tibia
CREM:
oligozoospermia
SNAI2:
decreased hematocrit
decreased hemoglobin content
hemorrhage
anemia
decreased body weight
decreased erythrocyte cell number
enlarged spleen
neonatal lethality, incomplete penetrance
abnormal blood vessel morphology
abnormal lung morphology
abnormal pulmonary alveolus morphology
decreased total body fat amount
increased blood urea nitrogen level
increased kidney weigh
thick pulmonary interalveolar septum
Blood Glucose
Body Height
NR4A1:
embryonic lethality during organogenesis
complete penetrance
impaired balance
Insulin-Secreting Cells
CREB3L1:
decreased body weight
decreased litter size
reduced male fertility
small testis
Body Height
Diabetes Mellitus, Type 2
C
GO:0060541: respiratory system development
R-HSA-400253: Circadian Clock
GO:0001944: vasculature development
GO:0000083: regulation of transcription involved in G1/S transition of mitotic cell cycle
GO:0071407: cellular response to organic cyclic compound
GO:0048701: embryonic cranial skeleton morphogenesis
GO:0009299: mRNA transcription
GO:0001706: endoderm formation
GO:0051124: synaptic growth at neuromuscular junction
GO:0003007: heart morphogenesis
GO:0010817: regulation of hormone levels
M65: PID FRA PATHWAY
GO:0007169: transmembrane receptor protein tyrosine kinase signaling pathway
R-HSA-3781860: Diseases associated with N-glycosylation of proteins
GO:0050673: epithelial cell proliferation
GO:0045936: negative regulation of phosphate metabolic process
GO:0001570: vasculogenesis
GO:1901615: organic hydroxy compound metabolic process
GO:0061061: muscle structure development
GO:0043393: regulation of protein binding
0 1 2 3 4 5 6 7 8
-log10(P)
Candidate positively selected snps around GPER1 in HUVEC
D
Chromosome 7
Gene
GPER1
Candidate snps
DNase-seq
H3K27ac
H3K4me1
H3K4me3
Elements

Supplementary Figure 10. Hypoxia oriented adaptation regulatory network and its functional enrichment. (A) Functional enrichment of *EPAS1* oriented network. Top terms include interleukin, oxidative stress, and cardiac muscle tissue growth, etc. (B) Hypoxia-oriented network (refer to Supplement Method for the network reconstruction). The size and shape meaning of nodes is the same as **Fig. 5**. (C) Functional enrichment of hypoxia oriented network. Top enriched terms are respiratory systems development, circadian clock, vasculature development, cell cycle (p-value<10^{-5}) and other terms. (D) Regulatory map for G protein-coupled estrogen receptor (*GPER1*), which is far from the *EPAS1* in the network. Source data are provided as a Source Data file.

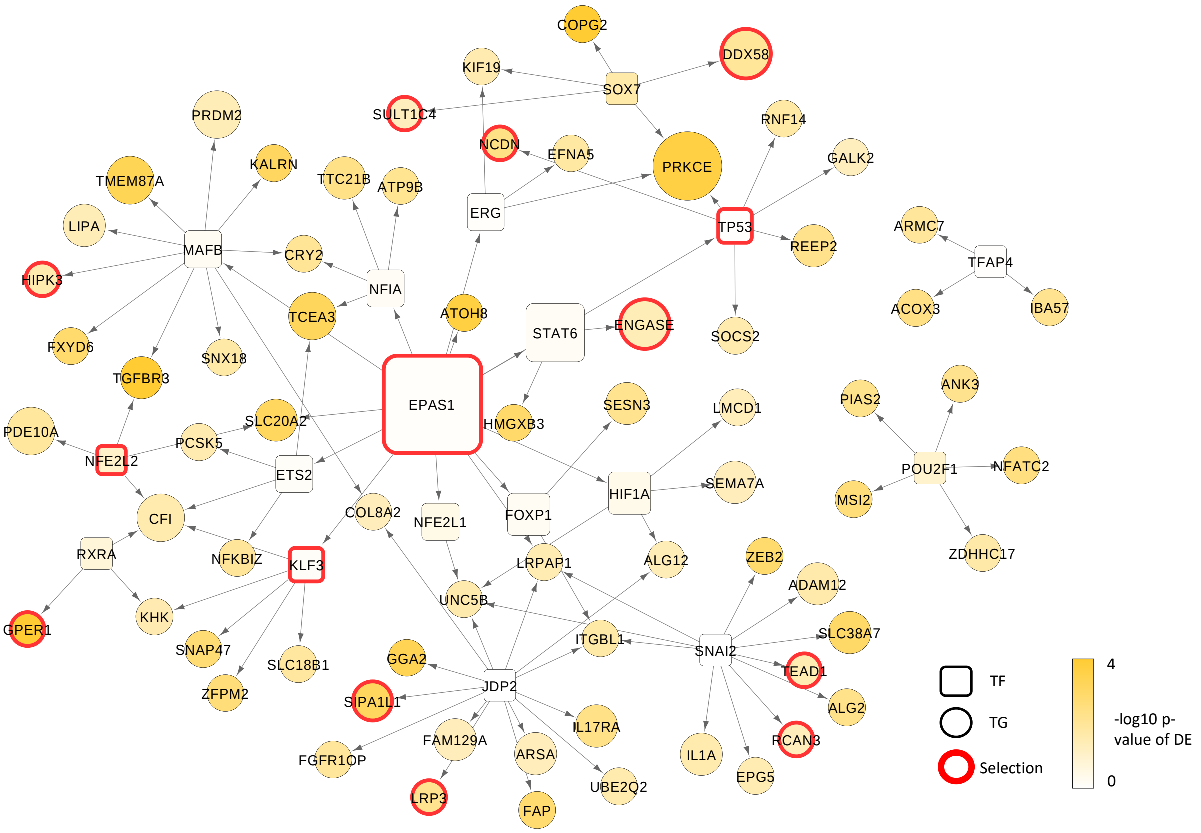

Supplementary Figure 11. 80 genes in the *EPAS1* oriented subnetwork are positively selected at least in one other organism of high-altitude adaptation. Node size is proportional to the number of organisms.

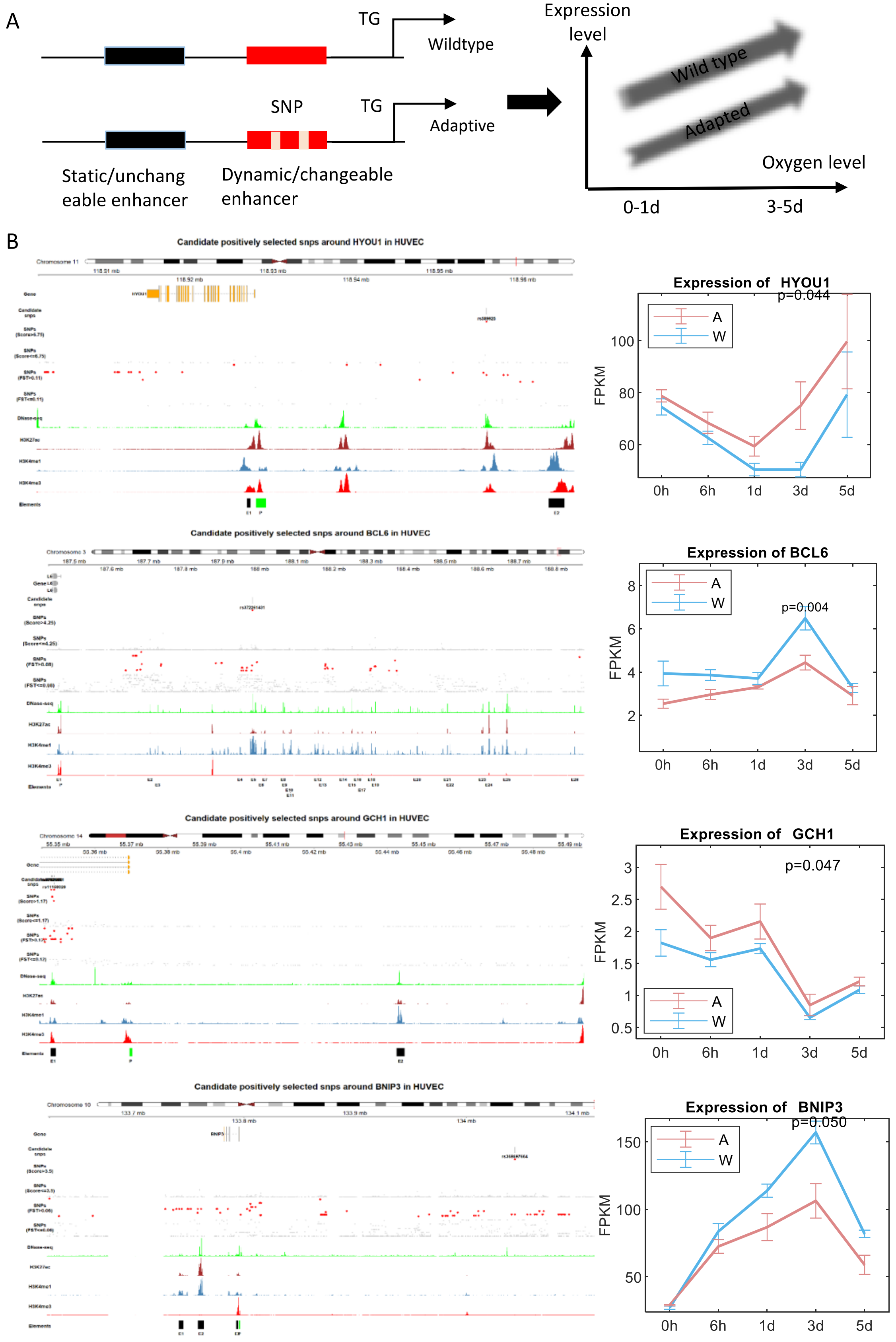
A
TG
Wildtype
SNP
TG
Adaptive
Static/unchangeable enhancer
Dynamic/changeable enhancer
Expression level
Wild type
Adapted
Oxygen level
0-1d
3-5d
B
Candidate positively selected snps around HYOU1 in HUVEC
Expression of HYOU1
p=0.044
FPKM
A
W
0h
6h
1d
3d
5d
Candidate positively selected snps around BCL6 in HUVEC
Expression of BCL6
p=0.004
Candidate positively selected snps around GCH1 in HUVEC
Expression of GCH1
p=0.047
Candidate positively selected snps around BNIP3 in HUVEC
Expression of BNIP3
p=0.050

Supplementary Figure 12. Combinatorial regulation model of active REs and active selected REs explains the blunted effect mechanism at the expression level. (A) Cartoon plot for the "Static/Unchangeable"(canonical enhancer) and "Dynamic/Changeable"(adapted enhancers) enhancers and their cooperation to generate down-regulation patterns. (B) Regulatory maps for *NOS3*, *BCL6*, *GCH1*, and *BNIP3* show blunted expression due to both active and active selected REs. The p-values were calculated by two-sided t-test (n=25 samples). Data are presented as mean values +/- standard error (n=5). Source data are provided as a Source Data file.

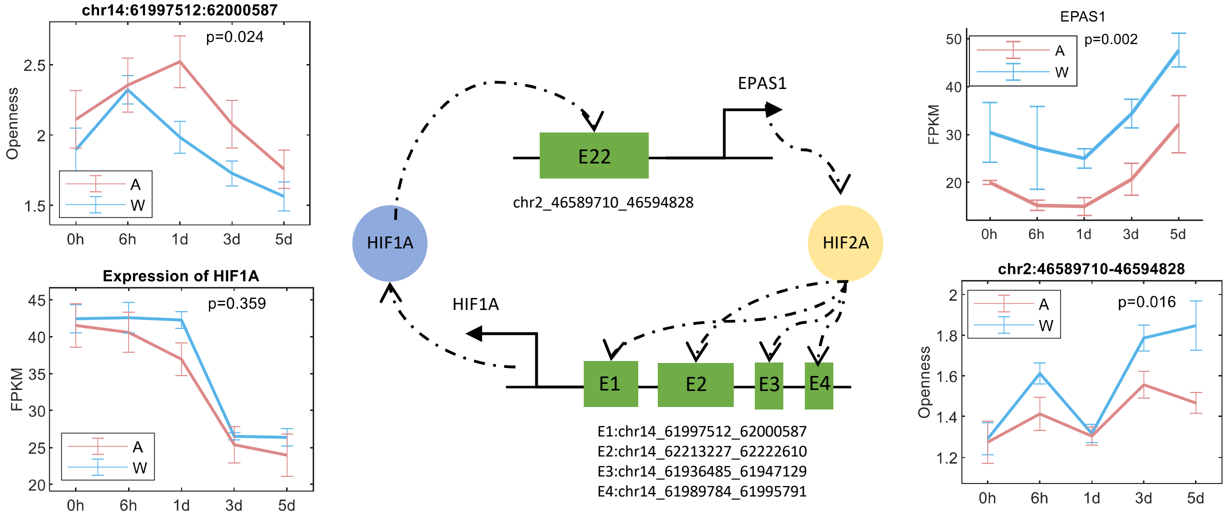

Supplementary Figure 13. Mutual feedback regulation of *HIF1A* and *EPAS1* via active REs and their dynamic expression pattern. The p-values were calculated by two-sided t-test (n=25 samples). Data are presented as mean values +/- standard error (n=5). Source data are provided as a Source Data file.

A

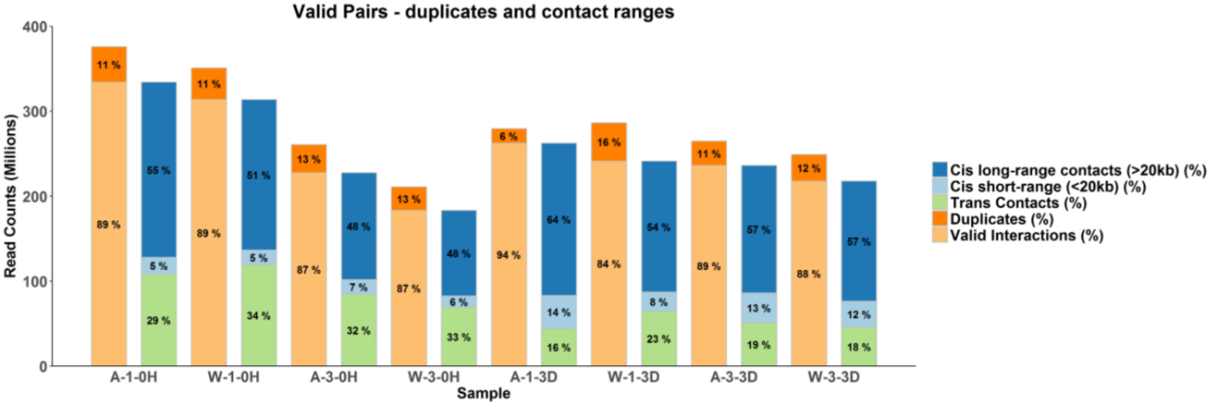

B

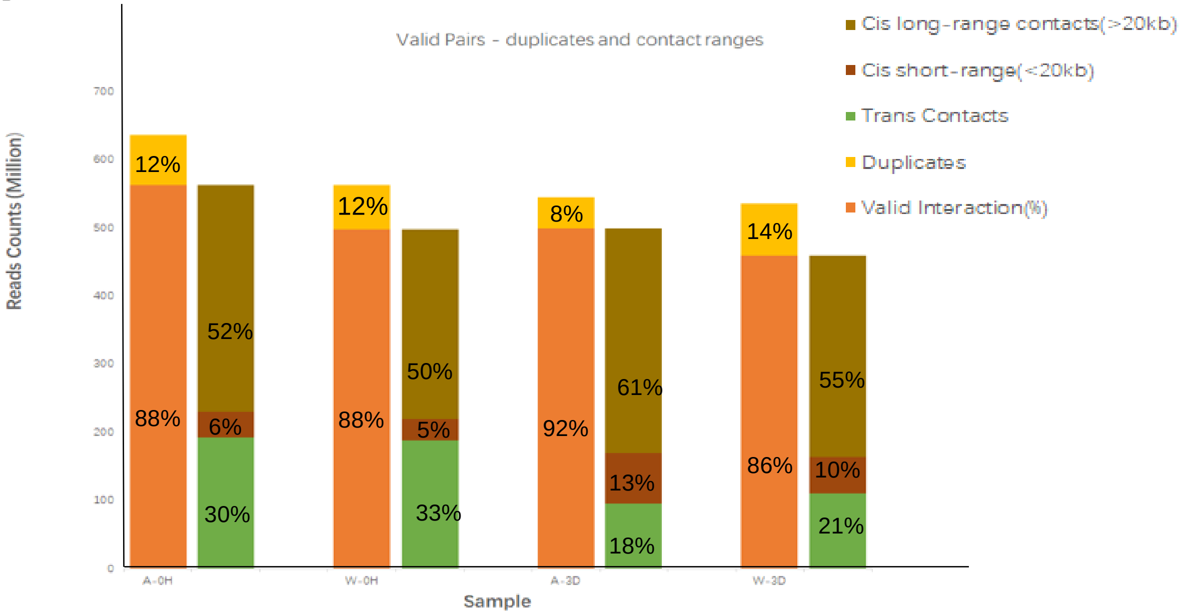

Supplementary Figure 14. High-quality Hi-C samples checked by the proportion of duplicate reads and different contact ranges. For each sample, we removed duplicated reads, and then divided remaining valid reads into cis long-range (>200k), cis short-range (<200k), and trans contacts, which are demonstrated by a different color. (A) The proportion of duplicate reads and contact ranges in 8 original samples. (B) The proportion of duplicate reads and contact ranges in 4 merged samples. For each population (Tibetan and Han) in each time point (0h and 3d), two replicates are merged together.

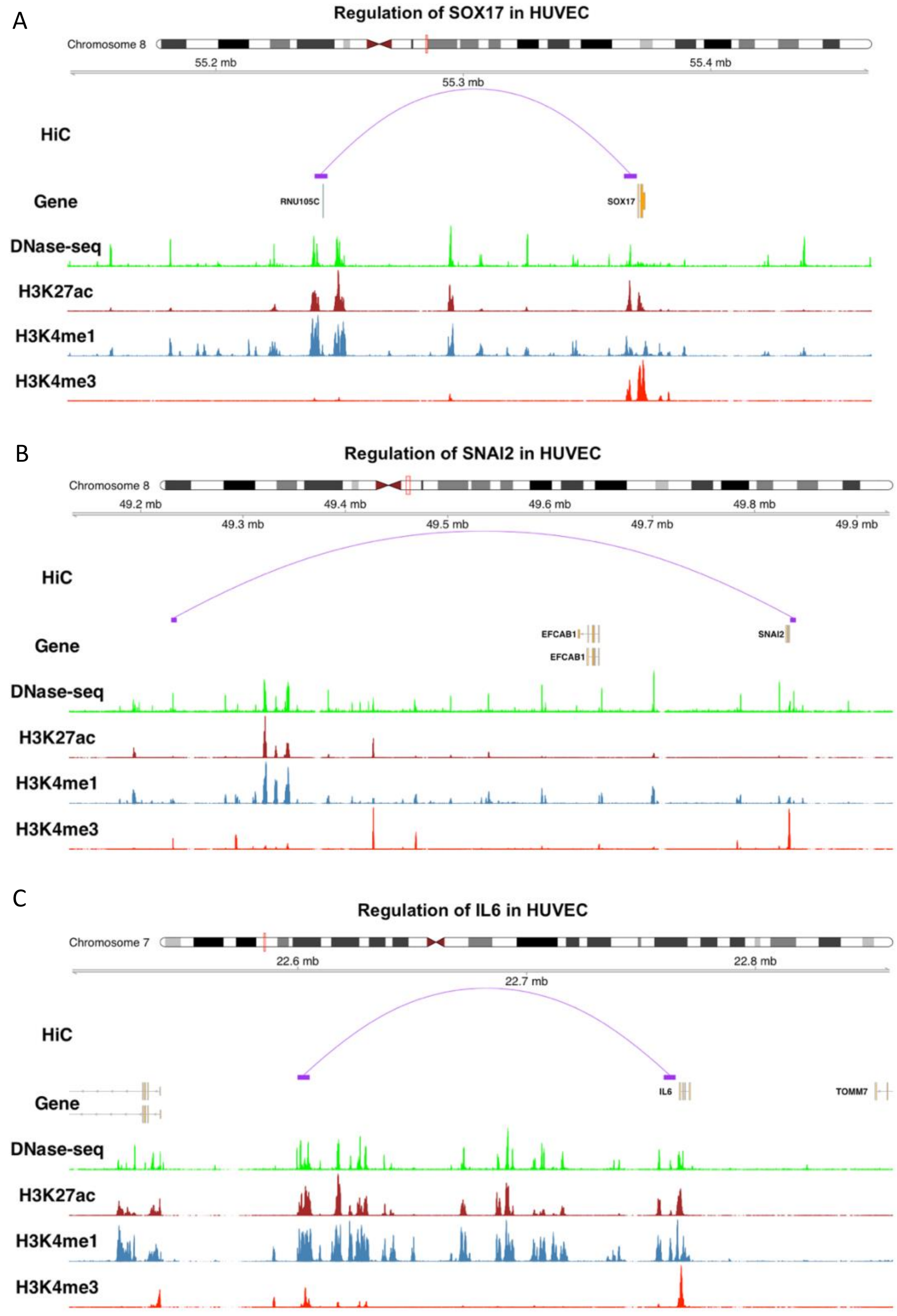

Supplementary Figure 15. Enhancer-promoter interactions detected by the Hi-C loops for (A) SOX17, (B) SNAI2, and (C) IL6.

A

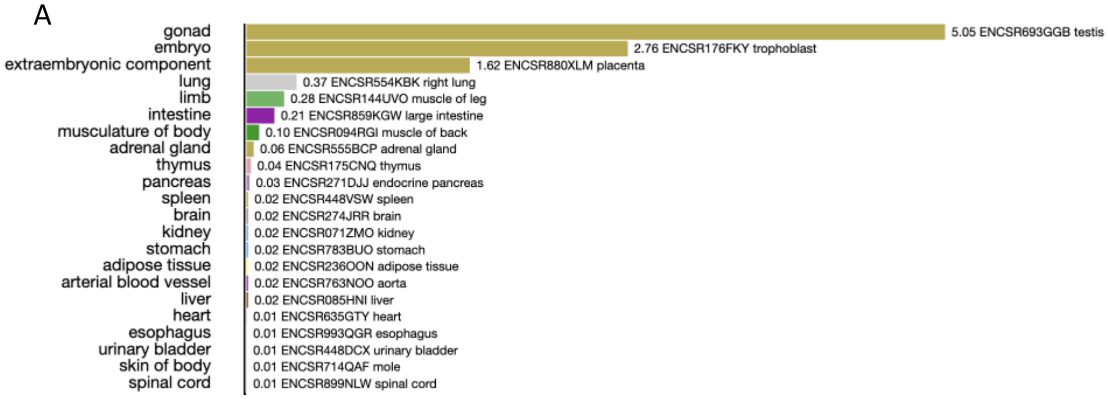
gonad
embryo
extraembryonic component
lung
limb
intestine
musculature of body
adrenal gland
thymus
pancreas
spleen
brain
kidney
stomach
adipose tissue
arterial blood vessel
liver
heart
esophagus
urinary bladder
skin of body
spinal cord
5.05 ENCSR693GGB testis
2.76 ENCSR176FKY trophoblast
1.62 ENCSR880XLM placenta
0.37 ENCSR554KBK right lung
0.28 ENCSR144UVO muscle of leg
0.21 ENCSR859KGW large intestine
0.10 ENCSR094RGI muscle of back
0.06 ENCSR555BCP adrenal gland
0.04 ENCSR175CNQ thymus
0.03 ENCSR271DJJ endocrine pancreas
0.02 ENCSR448VSW spleen
0.02 ENCSR274JRR brain
0.02 ENCSR071ZMO kidney
0.02 ENCSR783BUO stomach
0.02 ENCSR236OON adipose tissue
0.02 ENCSR763NOO aorta
0.02 ENCSR085HNI liver
0.01 ENCSR635GTY heart
0.01 ENCSR993QGR esophagus
0.01 ENCSR448DCX urinary bladder
0.01 ENCSR714QAF mole
0.01 ENCSR899NLW spinal cord

B

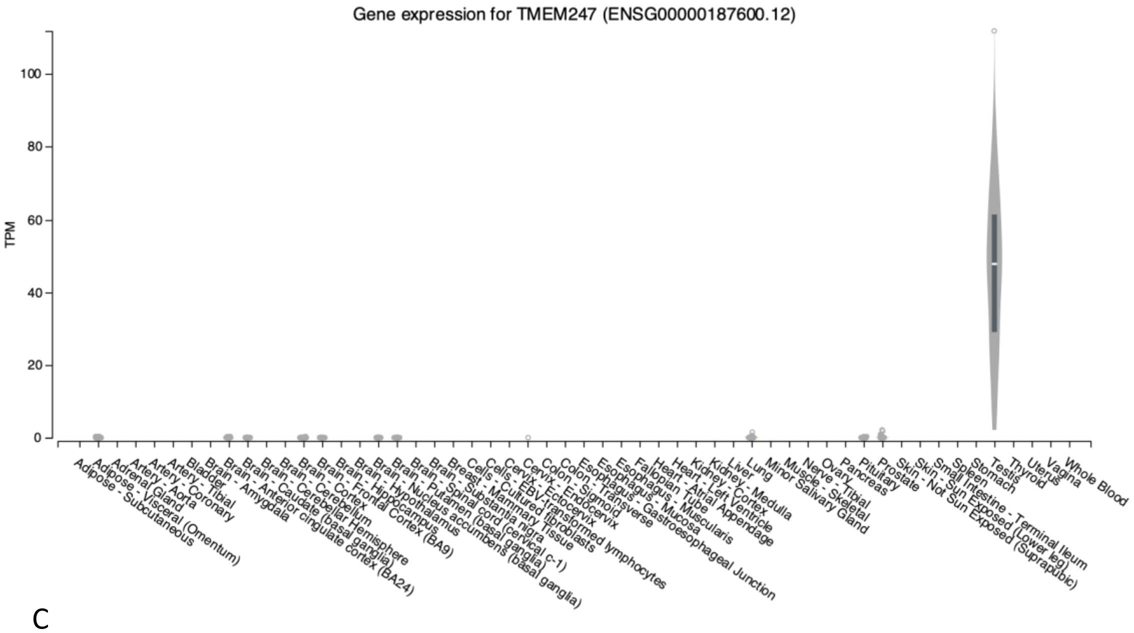
Gene expression for TMEM247 (ENSG00000187600.12)
TPM
0
20
40
60
80
100
Adipose - Subcutaneous
Adipose - Visceral (Omentum)
Adrenal Gland
Artery - Aorta
Artery - Coronary
Artery - Tibial
Bladder
Brain - Amygdala
Brain - Anterior cingulate cortex (BA24)
Brain - Caudate (basal ganglia)
Brain - Cerebellar Hemisphere
Brain - Cerebellum
Brain - Cortex
Brain - Frontal Cortex (BA9)
Brain - Hippocampus
Brain - Hypothalamus
Brain - Nucleus accumbens (basal ganglia)
Brain - Putamen (basal ganglia)
Brain - Spinal cord (cervical c-1)
Brain - Substantia nigra
Breast - Mammary Tissue
Cells - Cultured fibroblasts
Cells - EBV-transformed lymphocytes
Cervix - Ectocervix
Cervix - Endocervix
Colon - Sigmoid
Colon - Transverse
Esophagus - Gastroesophageal Junction
Esophagus - Mucosa
Esophagus - Muscularis
Fallopian Tube
Heart - Atrial Appendage
Heart - Left Ventricle
Kidney - Cortex
Kidney - Medulla
Liver
Lung
Minor Salivary Gland
Muscle - Skeletal
Nerve - Tibial
Ovary
Pancreas
Pituitary
Prostate
Skin - Not Sun Exposed (Suprapubic)
Skin - Sun Exposed (Lower leg)
Small Intestine - Terminal Ileum
Spleen
Stomach
Testis
Thyroid
Uterus
Vagina
Whole Blood

C

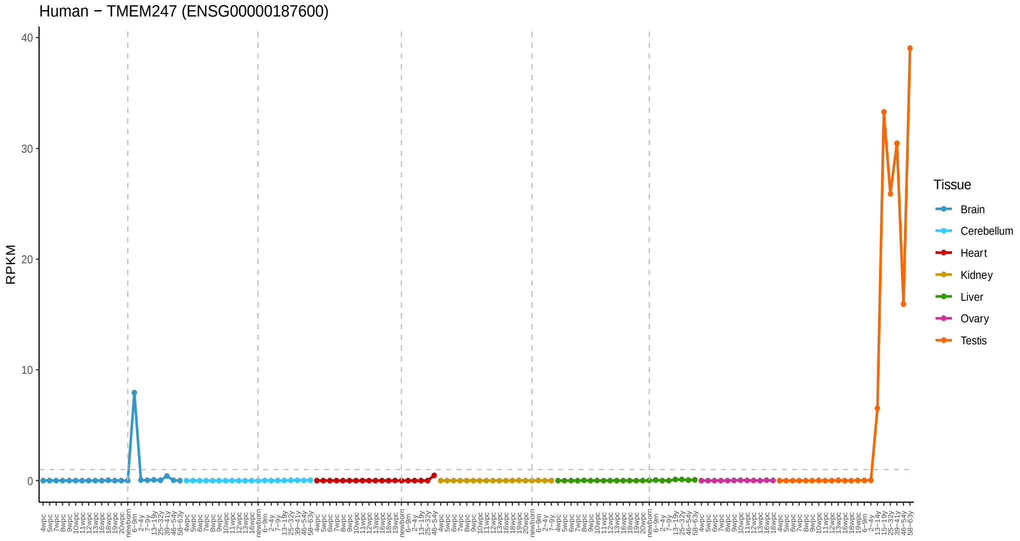
Human − TMEM247 (ENSG00000187600)
RPKM
0
10
20
30
40
Tissue
Brain
Cerebellum
Heart
Kidney
Liver
Ovary
Testis

Supplementary Figure 16. Expression profile of *TMEM247* in (A) ENCODE, (B) GTEx, and (C) developmental data across 6 major tissues in human [3]. From the above three datasets, TMEM247 is mainly expressed in testis, but not in other tissues or cell lines.

Supplementary References

1. Peng, Y. *et al.* Down-Regulation of EPAS1 Transcription and Genetic Adaptation of Tibetans to High-Altitude Hypoxia. *Mol Biol Evol* **34**, 818-830 (2017).
2. Azad, P. *et al.* High-altitude adaptation in humans: from genomics to integrative physiology. *J Mol Med (Berl)* **95**, 1269-1282 (2017).
3. Cardoso-Moreira, M. *et al.* Gene expression across mammalian organ development. *Nature* **571**, 505-509 (2019).
