# Supplementary material for: Chromatin accessibility landscape and regulatory network of high-altitude hypoxia adaptation
Source: Nat Commun. 2020 Oct 1;11:4928. doi: 10.1038/s41467-020-18638-8 (PMC7529806; doi:10.1038/s41467-020-18638-8)
Supplement: Supplementary file 3 — Description of Additional Supplementary Files [file 41467_2020_18638_MOESM3_ESM.pdf]

Description of Additional Supplementary Files

Supplementary Data 1. The summary of the high-quality ATAC-seq data for 50 samples.

Supplementary Data 2. List of the 1,647 active selected REs and their 1,146 target genes.

Supplementary Data 3. 7 active REs identified vPECA to regulate EPAS1's expression, the high Fst SNPs contained, and their regulatory strength by linear regression.

Supplementary Data 4. EPAS1's core regulatory network shows 621 TG are regulated by EPAS1 via 1,962 active REs.

Supplementary Data 5.
List of the 80 genes in the EPAS1 oriented network are positive selected at least in one other organism for high altitude adaption. The number of organisms and organism names are listed.

Supplementary Data 6. Statistics of 10 HUVEC WGS data.

Supplementary Data 7. Arguments of SNVs calling for 10 HUVEC WGS data.

Supplementary Data 8. The number of DEGs and DCAs in dynamics.

Supplementary Data 9. Functional enrichment analysis of DEGs. P-values were calculated by hypergeometric test with Benjamini-Hochberg correction.

Supplementary Data 10. Functional enrichment analysis of DCAs. P-values were calculated by hypergeometric test with Benjamini-Hochberg correction.

Supplementary Data 11. Tibetan- specific SNPs for ancestry characterization.
