# Supplementary material for: Chromatin accessibility landscape and regulatory network of high-altitude hypoxia adaptation
Source: Nat Commun. 2020 Oct 1;11:4928. doi: 10.1038/s41467-020-18638-8 (PMC7529806; doi:10.1038/s41467-020-18638-8)
Supplement: Supplementary file 15 — Reporting Summary [file 41467_2020_18638_MOESM15_ESM.pdf]

Corresponding author(s): Bing Su
Last updated by author(s): Jul 18, 2020

nature research

Reporting Summary

Nature Research wishes to improve the reproducibility of the work that we publish. This form provides structure for consistency and transparency in reporting. For further information on Nature Research policies, see Authors & Referees and the Editorial Policy Checklist .

Statistics

For all statistical analyses, confirm that the following items are present in the figure legend, table legend, main text, or Methods section.

n/a	Confirmed	
☐	☒	The exact sample size (*n*) for each experimental group/condition, given as a discrete number and unit of measurement
☐	☒	A statement on whether measurements were taken from distinct samples or whether the same sample was measured repeatedly
☐	☒	The statistical test(s) used AND whether they are one- or two-sided<br>*Only common tests should be described solely by name; describe more complex techniques in the Methods section.*
☒	☐	A description of all covariates tested
☐	☒	A description of any assumptions or corrections, such as tests of normality and adjustment for multiple comparisons
☐	☒	A full description of the statistical parameters including central tendency (e.g. means) or other basic estimates (e.g. regression coefficient) AND variation (e.g. standard deviation) or associated estimates of uncertainty (e.g. confidence intervals)
☐	☒	For null hypothesis testing, the test statistic (e.g. *F*, *t*, *r*) with confidence intervals, effect sizes, degrees of freedom and *P* value noted<br>*Give P values as exact values whenever suitable.*
☒	☐	For Bayesian analysis, information on the choice of priors and Markov chain Monte Carlo settings
☐	☒	For hierarchical and complex designs, identification of the appropriate level for tests and full reporting of outcomes
☐	☒	Estimates of effect sizes (e.g. Cohen's *d*, Pearson's *r*), indicating how they were calculated

Our web collection on statistics for biologists contains articles on many of the points above.

Software and code

Policy information about availability of computer code

Data collection	TopHat-2.0.13, Cufflinks-2.2.1.Linux_x86_64, Bowtie v1.0.0, Samtools.v0.1.19, Hotspot, Encode Chip-seq-pipeline-1.4, HiC-Pro_2.9.0.
Data analysis	vPECA matlab package (Matlab 2018a), HOMER v4.10, Cytoscape 3.71.

For manuscripts utilizing custom algorithms or software that are central to the research but not yet described in published literature, software must be made available to editors/reviewers. We strongly encourage code deposition in a community repository (e.g. GitHub). See the Nature Research guidelines for submitting code & software for further information.

Data

Policy information about availability of data

All manuscripts must include a data availability statement. This statement should provide the following information, where applicable:
- Accession codes, unique identifiers, or web links for publicly available datasets
- A list of figures that have associated raw data
- A description of any restrictions on data availability

The data generated in this study, including RNA-seq, ATAC-seq, and Hi-C data were deposited at [http://www.ncbi.nlm.nih.gov/geo/] (accession number: GSE145774) and Genome Sequence Achieve (project number: CRA002025; [https://bigd.big.ac.cn/gsa/browse/CRA002025]).
Source Data are provided in Zip folder.

Field-specific reporting

Please select the one below that is the best fit for your research. If you are not sure, read the appropriate sections before making your selection.

Life sciences study design

All studies must disclose on these points even when the disclosure is negative.

Sample size	No sample size calculation was performed. Usually for time series ATAC-seq and RNA-seq data, for instance in ENCODE, 2 replicates are performed to measure their consistency. In our study, 5 replicates for each time point in each population are sequenced.
Data exclusions	No data was excluded.
Replication	For RNA-seq and ATAC-seq data, we collected 5 biological replicates for each time point in each population. For Hi-C data, we collected 2 replicates for each time point in each population. For matched Whole-genome sequencing data, we have 5 replicates for each population.
Randomization	Umbilical cords were obtained from 131 normal, full term pregnancies at the People's Hospital of Lhasa, Tibetan. Then for all the samples we sequenced 4 positions based on EPAS1 and EGLN1's genotype, and divided the HUVECs into two groups adaptive vs. non-adaptive. Then we randomly selected 5 adaptive and 5 non-adaptive of HUVECs.
Blinding	The investigators were blinded to sample allocation during data collection and analysis.

Reporting for specific materials, systems and methods

We require information from authors about some types of materials, experimental systems and methods used in many studies. Here, indicate whether each material, system or method listed is relevant to your study. If you are not sure if a list item applies to your research, read the appropriate section before selecting a response.

Materials & experimental systems

n/a	Involved in the study	
☒	☐	Antibodies
☐	☒	Eukaryotic cell lines
☒	☐	Palaeontology
☒	☐	Animals and other organisms
☐	☒	Human research participants
☒	☐	Clinical data

Methods

n/a	Involved in the study	
☒	☐	ChIP-seq
☒	☐	Flow cytometry
☒	☐	MRI-based neuroimaging

Eukaryotic cell lines

Policy information about cell lines

Cell line source(s)	Human umbilical vein endothelial cells (HUVECs) from human umbilical cords.
Authentication	The HUVECs were isolated from the veins of human umbilical cord by the well-established method from "E. A. Jaffe, R. L. Nachman, C. G. Becker, C. R. Minick, Culture of human endothelial cells derived from umbilical veins. Identification by morphologic and immunologic criteria. J Clin Invest 52, 2745-2756 (1973)". Comparison of our data with ENCODE published data show their consistency.
Mycoplasma contamination	We used PCR to test if the cells were contaminated by mycoplasma.
Commonly misidentified lines (See ICLAC register)	*Name any commonly misidentified cell lines used in the study and provide a rationale for their use.*

Human research participants

Policy information about studies involving human research participants

Population characteristics	All the people involved in this study are 20-40 years old healthy women. No genetic relationships are reported between them. The ancestry of the donors was determined by the self-report and verified by the genetic analyses using genotyping and whole genome sequencing data. Only those Tibetans, whose lineal relatives are also Tibetan ethnicity within three generations, were included in this study. The same strategy was also implemented for Han donors.
Recruitment	Umbilical cords were obtained from 131 normal, full term pregnancies at the People's Hospital of Lhasa, Tibetan. Then for all the samples we sequenced 4 positions based on EPAS1 and EGLN1's genotype, and divided the HUVECs into two groups adaptive vs. non-adaptive. Then investigators randomly selected 5 adaptive and 5 non-adaptive of HUVECs. There is no self-selection bias in individual selection.
Ethics oversight	The protocol of this study was approved by the Institutional Review Board of the Kunming Institute of Zoology, Chinese Academy of Sciences.

Note that full information on the approval of the study protocol must also be provided in the manuscript.
